# Beyond BMI: Practical Guide for Clinicians to Integrate the Lancet Commission's Obesity Framework and King's Obesity Staging System

**DOI:** 10.1111/cob.70045

**Published:** 2025-09-09

**Authors:** Tak Ying Louise Ko, Alexander D. Miras, Dimitri J. Pournaras, Carel W. Le Roux

**Affiliations:** ^1^ Diabetes Complications Research Centre University College Dublin Dublin Ireland; ^2^ School of Medicine Ulster University Derry UK; ^3^ Department of Upper GI Surgery North Bristol NHS Trust Bristol UK; ^4^ Diabetes Research Centre Ulster University Coleraine UK

**Keywords:** beyond BMI, clinical obesity, health impact assessment, king's obesity staging criteria, obesity management

## Abstract

Body mass index (BMI) on its own is a poor diagnostic and staging tool for obesity because it does not measure health status. The newly published Lancet Clinical Obesity Criteria (LCOC) for defining clinical obesity distinguish preclinical and clinical obesity based on organ or tissue dysfunction. The King's Obesity Staging System (KOSS) goes further and incorporates biomedical, psychosocial, and economic factors while offering a practical, holistic, and health domain‐specific assessment of obesity's impact. This paper compares and maps the LCOC against the KOSS to highlight their complementary aspects, strengths, and potential for integration. By combining the LCOC philosophical framework with the practical patient‐centred approach of the KOSS, we propose a unified model that enhances diagnostic ability and allows the clinician to track the impact of any obesity treatment. This integrated framework advances obesity management, addressing both medical, functional, and broader psychosocial challenges.

## 1 Introduction

While BMI is a convenient tool and historical standard for obesity diagnosis, it is limited in its ability to accurately capture the full complexity of obesity's impact on individual health [1]. Recognising the shortcomings of BMI as a sole diagnostic tool, new frameworks have emerged to offer a more nuanced and individualised approach to obesity assessment. Among these are the Lancet Clinical Obesity Criteria (LCOC) [2] and the King's Obesity Staging System (KOSS) [3], both of which aim to address the gaps left by BMI by considering additional factors, such as functional health, organ dysfunction, and psychosocial impact. Other approaches to staging obesity include Obesity Surgery Score, Obesity Surgery Mortality Risk Score, and Edmonton Obesity Staging System (EOSS) [4, 5]. In particular, the EOSS, introduced in 2009, is the most studied and marked a significant departure from anthropometric classification alone [5] and has demonstrated utility in guiding clinical decision‐making beyond using BMI alone [4]. The KOSS takes this further by segregating the symptoms of clinical obesity into distinct domains to allow for more accurate staging [3]. Together, these now represent an important step toward a more comprehensive, patient‐centred approach to obesity management, moving away from a sole focus on weight and toward a broader evaluation of health outcomes.

One of the reasons BMI has remained a widely used screening tool is due to its simplicity and objectivity [1]. In contrast, the LCOC framework necessitates objective evidence of organ dysfunction or functional impairment to diagnose clinical obesity [2]. However, the binary distinction between preclinical and clinical obesity introduces inherent subjectivity, particularly in cases where metabolic dysfunction exists on a continuum rather than a discrete disease state. Conditions such as heart failure with preserved ejection fraction or metabolic dysfunction‐associated steatohepatitis (MASH) are clearly pathological and warrant classification as ‘clinical obesity’ [2], but conditions such as prediabetes or metabolic dysfunction‐associated steatotic liver disease (MASLD) pose a diagnostic grey area, leading to potential inconsistencies in classification across healthcare providers without being able to account for the variability in symptom presentation. This variability may complicate the integration of LCOC into routine practice, as clinicians may differ in their interpretation of what constitutes clinically significant dysfunction. Since functional impairments and organ dysfunction are central to defining clinical obesity, their diagnostic thresholds must be clearly established and standardised to prevent inconsistencies in clinical decision‐making. To address these challenges, integrating LCOC's organ dysfunction‐based diagnostic framework with the domain‐specific staging of KOSS offers a more refined stratification of obesity severity. This complementary approach ensures that obesity diagnosis and management are comprehensive and sensitive to the heterogeneity of the presentation of obesity and allows for obesity to be staged on the continuum of severity in which it presents itself.

## 2 LCOC: A CHRONIC, Systemic Disease Framework

The LCOC [2] defines obesity as a chronic, systemic disease with two stages: preclinical and clinical obesity. Clinical obesity is diagnosed when a person has confirmed excess adiposity and meets at least one of 18 criteria across 11 body systems and one functional domain, indicating organ or tissue dysfunction caused by adiposity. In contrast, preclinical obesity refers to excess adiposity without evident dysfunction. This distinction helps identify clinically significant complications while avoiding overdiagnosis in individuals who may have increased body fat without associated health complications. However, applying the LCOC in practice is challenging—especially for non‐specialists—as it requires detailed assessments beyond BMI, including anthropometric, metabolic, and organ function evaluations.

## 3 KOSS: A Comprehensive, Domain‐Specific Approach

While the LCOC defines obesity through organ dysfunction, the KOSS offers a broader, multidimensional framework that assesses obesity across 15 domains—including metabolic, functional, psychosocial, and economic factors [3]. Each domain is scored from 0 (normal) to 3 (advanced dysfunction), capturing a wide range of obesity‐related impacts. By including domains beyond only bodyweight, the KOSS recognises that obesity affects far more than just body mass. It acknowledges the role of obesity in functional health impairments such as reduced mobility, cardiovascular complications, and chronic pain, as well as the psychological and social burdens, including body image issues, stigma, and the financial strain of managing obesity. It also enables clinicians to track health improvements over time, showing how interventions affect overall health—not just weight—by highlighting domain‐specific changes in obesity treatment even when weight is unchanged [3].

## 4 Integrating LCOC and KOSS

Integrating the LCOC and KOSS offers a more dynamic and practical approach to obesity diagnosis and management (Table 1). The LCOC provides a clear, objective diagnostic framework by identifying obesity based on organ dysfunction and tissue damage, ensuring that individuals with clinically significant obesity are accurately diagnosed. The KOSS, on the other hand, offers a broader, multidimensional perspective on obesity's impact, capturing the full range of physical, psychological, and social consequences. By combining the strengths of both systems, clinicians can gain a more comprehensive understanding of an individual's health and provide more tailored, effective interventions.

**TABLE 1:** LCOC‐KOSS integrated model.

	Stage 0 (no problem, Equivalent to no organ or tissue dysfunction)	Stage 1 (equivalent to alteration in organ structure but no organ dysfunction)	Stage 2 (equivalent to alteration in organ function)	Stage 3 (equivalent to severe organ dysfunction)
Airway	Normal	Snoring	Apnoea or hypopnoea during sleep Hypoventilation, breathlessness, wheezing	Respiratory failure Cor pulmonale
Body mass and adiposity	BMI < 25	Waist circumference < 102 cm (men) < 88 cm (women) Waist‐to‐hip ratio < 0.9 (men) < 0.85 (women) Waist‐to‐height ration < 0.5	Waist circumference > 102 cm (men) > 88 cm (women) Waist‐to‐hip ratio > 0.9 (men) > 0.85 (women) Waist‐to‐height ration > 0.5	BMI > 40
Cardiovascular disease	< 10% risk of major adverse cardiovascular event	10%–25% risk of major adverse cardiovascular event	Recurrent deep‐vein thrombosis or pulmonary embolism Raised arterial blood pressure Ischaemic heart disease or stroke Chronic or recurrent atrial fibrillation	Heart failure with reduced ejection fraction (due to reduced left ventricular systolic function) Chronic fatigue and lower limb oedema (due to impaired diastolic function—heart failure with preserved ejection fraction) Pulmonary artery hypertension
Diabetes	Normo‐glycaemia	Non‐diabetic hyperglycaemia	Hyperglycaemia, high triglyceride levels, and low HDL cholesterol	Complications of diabetes
Economic	No impact	High cost of living (e.g., expensive clothes, travel)	Workplace discrimination	Unemployment due to obesity
Functional	Can manage three flights of stairs Normal Joint Function	Manages one to two flights of stairs Joint discomfort, Reduced Flexibility	Manage less than 1 flight of stairs Chronic, severe knee or hip pain (associated with joint stiffness and reduced range of motion) Substantial, age‐adjusted limitations of daily living Osteoarthritis	Lower limb lymphoedema (causing chronic pain, reduced range of motion, or both) House bound Disabling Osteoarthritis
Gonadal	Normal sexual and/or reproductive function	Hirsutism Acne Alopecia Low libido	Anovulation, oligomenorrhea and polycystic ovary syndrome, Male hypogonadism Erectile dysfunction	Infertility
Health status perceived (mental health)	Normal	Low mood or QoL	Mental illness (e.g., depression, anxiety)	Unstable or resistant mental illness
Image (body)	Normal	Dislikes body image	Body image dysphoria Disordered eating	Eating disorder due to body image dysphoria
Junction of the gastro‐oesophagus	Normal	Infrequent indigestion, heartburn	Gastro‐oesophageal reflux disease	Barrett's oesophagus, aspiration
Kidney	Normal	Microalbuminuria with preserved eGFR	Chronic urinary incontinence Microalbuminuria with chronic kidney disease stage 2	Chronic kidney disease stage 3–5
Liver	Normal	Metabolic dysfunction‐associated steatotic liver disease without fibrosis (MASLD)	Metabolic dysfunction‐associated steatotic liver disease with fibrosis (MASH)	Cirrhosis
Medication	None	Easy to manage medication	Polypharmacy	Complicated polypharmacy
Neurology	Normal	Headache	Vision loss, recurrent headaches, or both (due to raised intracranial pressure)	Neurological deficiency
Other	Normal organ function	Impaired organ structure	Organ dysfunction	Severe organ dysfunction

In an integrated model, the LCOC's stages of preclinical and clinical obesity can be mapped onto the KOSS's domain‐based scoring system. Stages 0 and 1 of the KOSS correspond to preclinical obesity, while stages 2 and 3 align with clinical obesity as defined by the LCOC. This integration allows for more precise tracking of treatment progress, as clinicians can evaluate improvements in various KOSS domains, even if a patient's weight does not change significantly. For example, a patient who loses only a small amount of weight may still show significant improvements in functional health, such as reduced joint pain or improved mobility, while another patient with substantial weight loss may show no change in body image perception, which can be captured through the KOSS framework. This comprehensive approach ensures that obesity treatment focuses on improving overall health and quality of life rather than simply achieving weight loss.

## 5 Addressing Psychosocial and Economic Impacts

One of the key strengths of the KOSS is its inclusion of psychosocial and economic factors, such as health status perceived, image, and the financial burden of obesity. These elements are often overlooked in traditional obesity management but are crucial for understanding the full impact of obesity on an individual's life. The LCOC framework, in contrast, focuses primarily on the physiological aspects of obesity, such as organ or tissue dysfunction. While the 18 obesity‐related complications defined by the LCOC are critical for supporting the diagnosis of clinical obesity, they do not explicitly account for the psychosocial and economic dimensions of the disease. By integrating the KOSS's broader assessment with the LCOC's clinical criteria, clinicians can better address the full spectrum of obesity's impact, leading to more personalised and compassionate care.

## 6 Comparison With the EOSS

While the EOSS was a major advance in moving beyond BMI by incorporating metabolic, functional, and mental parameters, it remains limited by its more biomedical focus and fewer staging domains. EOSS primarily evaluates comorbidities and functional limitations to stratify risk, but does not offer the granularity or multidimensional assessment found in the KOSS. In contrast, KOSS incorporates a broader spectrum of obesity‐related impacts, including economic burden, body image, mental health, and perceived health status, which are crucial in holistic obesity care. KOSS also allows for more sensitive tracking of treatment outcomes across multiple health domains, even in the absence of significant weight loss, while EOSS is better suited to initial risk stratification and diagnosis. By integrating the KOSS with LCOC, this proposed model addresses both the diagnostic clarity of LCOC and the comprehensive patient‐centred staging of KOSS, offering a more functional and actionable approach than EOSS alone.

## 7 Discussion of Integration

We have integrated two distinct models for obesity assessment—LCOC and KOSS—into a multidimensional framework that enables structured staging of obesity along a continuum rather than a binary disease/no‐disease paradigm. Clinically, this model establishes a standardised framework for staging obesity, ensuring consistent and precise communication among healthcare providers and researchers. Traditionally, the term ‘obesity’ lacked specificity, offering little context regarding disease severity or associated comorbidities [5]. By introducing a staging system into the LCOC definition, this model provides a clear, structured means of categorising the extent and impact of obesity that reflects the unique presentation and severity of obesity in each patient.

The integration of the LCOC and KOSS offers several benefits for clinicians and patients alike. For clinicians, the combined framework provides a practical, comprehensive, and individualised approach to obesity management, enabling them to diagnose obesity more accurately, track treatment progress across multiple domains, and address both the physiological and psychosocial aspects of the condition. For patients, this integrated approach reduces the focus on weight loss and helps to mitigate the stigma often associated with obesity. By recognising obesity as a multifactorial disease, this model fosters a more compassionate and patient‐centred approach to care.

Furthermore, this approach aligns with the LCOC recommendations and consensus that ‘staging systems for clinical obesity, reflecting the effect of a disease on quality of life and prognosis, are necessary to facilitate treatment choices and should be the focus of future work’, even though the LCOC did not propose a staging system itself [2]. With current knowledge, it remains unclear what degree of weight loss is necessary to achieve specific therapeutic goals, as different clinical manifestations of obesity—cardiovascular, metabolic, and musculoskeletal—require varying treatment intensities and respond differently to weight reduction [2, 6]. Moreover, as with any disease management, the success of obesity treatment should be defined by measurable improvements in clinical outcomes, rather than relying solely on surrogate markers of risk or weight reduction itself [2, 6]. Thus, our proposed LCOC‐KOSS integrated model builds upon and advances this existing consensus by providing a structured, clinically meaningful framework.

Additionally, this proposed integration considers the quality of life of the patient including the psychological and economic impacts of the disease of obesity. Historically, the psychological impact of obesity has been underrecognized in clinical practice, despite its well‐documented bidirectional relationship with disease progression [2, 7]. The alienation and objectification frequently reported by individuals with obesity can negatively influence self‐perception, health behaviours, and engagement with medical care, further exacerbating disease progression [8]. Furthermore, weight discrimination is reported in up to 42% of patients with obesity [2]. The burden of this is mitigated with this approach by ensuring the psychosocial burdens of obesity are recognised, assessed, and tracked accordingly.

However, there are some challenges to integrating these two frameworks. One of the primary challenges is the inherent subjectivity associated with certain domains of the KOSS, particularly those related to psychosocial and economic factors, such as body image and perceived health status. These factors rely heavily on self‐reported measures and clinician judgement, which can introduce variability in assessment. However, Aasheim et al. also documented that across a small sample size of 11 clinicians, inter‐observer variability using KOSS was low [3]. To further mitigate this, the use of validated patient‐reported outcome measures (PROMs) could help standardise assessments and improve the reliability of data collection in the future.

Another challenge in staging obesity using the LCOC‐KOSS integrated model is its inability to account for irreversible conditions, particularly in cases of recurrent deep‐vein thrombosis, pulmonary embolism, ischaemic heart disease, or stroke. These conditions, once diagnosed, establish a permanent medical history, meaning that even with successful treatment or risk reduction, a patient's staging cannot be downgraded and their cardiovascular domain remains at stage 2. This creates a limitation in the model's ability to reflect improvements in health status over time—one of the aspects in obesity management it seeks to improve. Staging systems, by definition, aim to stratify disease severity and provide a framework for clinical decision‐making, risk stratification, and treatment planning. However, certain chronic or past medical events, such as cardiovascular disease or thromboembolic events, do not necessarily improve or disappear with intervention. For example, a patient with recurrent DVT or PE will always have a history of venous thromboembolism, even if anticoagulation therapy or lifestyle changes significantly reduce their risk of recurrence. In such cases, our staging model does not fully capture the dynamic nature of obesity treatment and disease modification, as patients who successfully mitigate their health risks are not able to ‘move down’ a stage, despite substantial health improvements. From a clinical perspective, a staging model that does not allow for downgrading may lead to overestimation of current disease burden in patients who have effectively managed their conditions. However, the vast majority of this model largely reflects dynamic measures that can demonstrate either improvements or deterioration in the disease of obesity.

Another limitation of the LCOC‐KOSS integrated model is its reliance on the domain ‘Other’ to capture a multitude of obesity‐related complications including dermatological complications of obesity [9]. Obesity‐related skin complications arise from skin‐on‐skin friction, moisture retention, and mechanical strain, leading to intertrigo, bacterial and fungal infections, and delayed wound healing and conditions such as cellulitis, stasis dermatitis, and hidradenitis suppurativa [2, 9].

Another challenge is that the LCOC and KOSS were developed independently, and aligning them required subjective mapping of KOSS stages onto the LCOC's definitions of preclinical and clinical obesity. While this mapping is clinically intuitive, it has yet to be validated in real‐world settings. Additionally, the lack of longitudinal data on the integrated model's effectiveness in improving patient outcomes is a limitation. While both frameworks have shown clinical utility, more research is needed to determine the long‐term impact of the integrated model on obesity management. Future directions in research may include prospective validation of the integrated LCOC and KOSS, comparative studies with the EOSS, and qualitative studies to assess the acceptability by different healthcare professional groups. Similar studies have been done on the clinical utility of emerging staging systems, especially with EOSS [4], and can be instrumental in validating and refining assessment methods of obesity.

## 8 Conclusion

The integration of the LCOC and KOSS represents a significant advancement in the management of obesity. By combining the LCOC's focus on organ dysfunction with the KOSS's multidimensional approach, clinicians can gain a more comprehensive understanding of obesity's impact on each patient. This integrated model shifts the focus of obesity management from weight loss alone to a more holistic evaluation of health outcomes, improving diagnostic accuracy, tracking meaningful health improvements, and reducing stigma. As obesity is increasingly recognised as a chronic disease that requires long‐term management, this integrated framework offers a practical, patient‐centred approach to diagnosis and treatment, ultimately improving the quality of care for people with the disease of obesity.

## Conflicts of Interest

C.W.L.R. reports grants from the Irish Research Council, Science Foundation Ireland, Anabio, and the Health Research Board. He serves on advisory boards of Novo Nordisk, Herbalife, GI Dynamics, Eli Lilly, Johnson & Johnson, Glia, Keyron, and Boehringer Ingelheim. C.W.L.R. is a member of the Irish Society for Nutrition and Metabolism outside the area of work commented on here. He was the chief medical officer and director of the Medical Device Division of Keyron in 2021. Both of these are unremunerated positions. No patients have been included in any of Keyron's studies and they are not listed on the stock market. C.W.L.R. was gifted stock holdings in September 2021 and divested all stock holdings in Keyron in September 2021. He continues to provide scientific advice to Keyron for no remuneration. D.J.P. has been funded by the Royal College of Surgeons of England. He receives consulting fees from GSK, Johnson & Johnson and Novo Nordisk, and payments for lectures, presentations and educational events from Johnson & Johnson, Medtronic, Novo Nordisk, Pfizer and Sandoz. A.D.M. has received research funding from the European Union, Medical Research Council (MRC), National Institute for Health and Care Research (NIHR), HSC R&D division, Jon Moulton Charitable Foundation, Anabio, Fractyl, Boehringer Ingelheim, Eli Lilly, Gila, Randox, and Novo Nordisk. A.D.M. has received honoraria for lectures and presentations from Novo Nordisk, AstraZeneca, Currax Pharmaceuticals, Boehringer Ingelheim, Screen Health, GI Dynamics, Algorithm, Eli Lilly, Ethicon, and Medtronic. A.D.M. is a shareholder in the Beyond BMI clinic, which provides clinical obesity care. Tak Ying Louise Ko has no conflicting interests to declare that may influence their judgements on what is published.

## Data Availability

Data sharing is not applicable to this article as no new data were created or analysed in this study.
